# The Role of Optimism and Psychosocial Factors in Athletes Recovery From ACL Injury: A Longitudinal Study

**DOI:** 10.3389/fspor.2020.00116

**Published:** 2020-10-02

**Authors:** Tom Williams, Lynne Evans, Angus Robertson, Lew Hardy, Stuart Roy, Daniel Lewis, Freya Glendinning

**Affiliations:** ^1^School of Health, Sport and Professional Practice, University of South Wales, Pontypridd, United Kingdom; ^2^School of Sport and Health Sciences, Cardiff Metropolitan University, Cardiff, United Kingdom; ^3^Cardiff Sports Orthopaedics LLP, Spire Cardiff Hospital, Cardiff, United Kingdom; ^4^School of Sport, Health and Exercise Sciences, Bangor University, Gwynedd, United Kingdom

**Keywords:** optimism, psychosocial factors, rehabilitation adherence, perceived knee function, ACL, Bayesian structural equation modeling

## Abstract

Despite a growing interest into the role of psychosocial factors during the recovery period following sports injuries, there remains a paucity of longitudinal studies examining the indirect relationships between psychosocial factors, psychological responses, and recovery outcomes. The purpose of this study was to construct and test a conceptual model which examined the indirect relationships between optimism, psychosocial factors, rehabilitation adherence, and perceived knee function up to 12 months post anterior cruciate ligament (ACL) surgery. A prospective, longitudinal, and repeated measures design was employed, wherein 81 injured athletes (*M*_*age*_ 26.89, *SD* = 7.52) completed measures of optimism, psychosocial factors, rehabilitation adherence, and perceived knee function on seven occasions (pre-surgery to 1 year post-surgery). Bayesian structural equation modeling evaluated the hypothesized indirect relationships proposed within the conceptual model. The main findings from this study was empirical support for a time-ordered, conceptual model which demonstrated that pre-surgery optimism had a significant overall indirect effect on perceived knee function at 12 months post-surgery (*sum of indirect*; αβ = 0.08, post. *SD* = 0.05, CI [0.01, 0.04]), as well as a specific indirect effect through secondary appraisal at 1 month post-surgery, efficacy at 2 months post-surgery, and rehabilitation adherence at 6 months post-surgery (αβ = 0.03, post. *SD* = 0.03, CI [0.00, 0.10]). Collectively, this study provides support for a number of previously hypothesized, but not empirically examined, indirect relationships between optimism, psychosocial factors and recovery outcomes. In doing so, we provide a conceptual model which has the potential to help guide individualized treatment recommendations, as well as identify individuals at risk of compromised recovery outcomes following ACL surgery.

## Introduction

A rupture to the anterior cruciate ligament (ACL) is one of the most common and costly sports injuries sustained by athletes that not only threatens their ability to return-to-sport (Lai et al., 2017), but increases their risk of a subsequent injury (Nagelli and Hewett, 2017), and for many impairs their long-term quality of life (Filbay et al., 2017). It also places a huge financial burden on the healthcare system, with estimated hospital costs of over A$75 million (€45 million) per year in Australia alone (Janssen et al., 2011). Perhaps not surprisingly, therefore, the increased incidence of ACL injuries over the last 20 years (Herzog et al., 2017) has been marked by a concomitant rise in the research attention afforded to factors associated with improving ACL outcomes—including those of a psychosocial nature (e.g., Brewer, 2010; Wadey et al., 2014; Ardern et al., 2015).

Much of this empirical research has been underpinned by either Wiese-Bjornstal et al.'s (1998) integrated model of psychological response to sport injury and it's various iterations (Wiese-Bjornstal et al., 1995; Wiese-Bjornstal, 2010), or the biopsychosocial model of sport injury rehabilitation (Brewer et al., 2002). The integrated model of psychological response suggests pre-injury factors (e.g., personality traits), and post-injury personal (e.g., self-motivation) and situational (e.g., treatment environment) factors moderate an injured athletes' cognitive (e.g., attributions), emotional (e.g., distress), and behavioral (e.g., adherence to rehabilitation) responses, mediated by the process of cognitive appraisal. In the biopsychosocial model, psychological factors (personality, affect, behavior, cognition) are proposed to have direct relationships with intermediate recovery outcomes (e.g., strength, pain, joint laxity), and sport injury recovery outcomes (e.g., functional performance, quality of life, readiness to return-to-sport). They are also postulated to have indirect relationships with intermediate outcomes, via biological factors (e.g., tissue repair, immune functioning), and with sport injury recovery outcomes, via intermediate outcomes. Although these models have yet to be examined in their entirety, researchers have provided empirical support for a number of their central tenets, including the impact of personality, affect, behavior, and cognitions on post-injury psychological responses (for a review see Brewer and Redmond, 2017), and to a lesser extent, sport injury rehabilitation outcomes (for a review see Brewer, 2010). Of these variables, those related to outcome-expectancy appear to hold most promise in trying to understand the complex relationships between psychosocial factors, psychological responses, and recovery outcomes (Everhart et al., 2015).

Arguably, one of most salient outcome-expectancy variables is dispositional optimism. A personality trait that reflects the extent to which people hold favorable expectancies for their future (Carver et al., 2010), dispositional optimism has been associated with faster healing rates (Ievleva and Orlick, 1991), lower incidence of injury, and better psychological adjustment following injury (Wadey et al., 2013). These beneficial outcomes appear to result from differences in athletes' health-promoting behaviors, including the coping strategies they adopt in stressful situations (Solberg Nes and Segerstrom, 2006), and the extent to which they engage in rehabilitation (Wadey et al., 2013). Optimists typically employ approach-focused coping strategies aimed at eliminating, reducing, or managing stressors and emotions (Schwarzer et al., 2005), which mediate negative psychological responses and positively correlate with rehabilitation adherence (Udry, 1997; Scherzer et al., 2001; Wadey et al., 2013). Pessimists, on the other hand, typically employ avoidance coping strategies such as seeking to ignore, avoid, or withdraw from stressors (Solberg Nes and Segerstrom, 2006; Carver et al., 2010), which have been associated with lower rehabilitation adherence (Udry, 1997), increased levels of distress (Blalock and Joiner, 2000), and greater knee pain (Rosenberger et al., 2004).

These differential coping profiles are proposed to be a function of differences in their appraisals of the stressor (Chang, 1998). According to Lazarus and Folkman (1984), two types of cognitive appraisals occur in response to stressors: primary and secondary. During primary appraisals, stressors are assessed with regard to the implications for an individual's well-being. Secondary appraisals involve an assessment of what coping strategies are available, the likelihood of a strategy being successful, and the likelihood one can apply a particular strategy or set of strategies. Research suggests optimists and pessimists make similar primary appraisals, but differ in their secondary appraisals, with pessimists perceiving less control over their ability to cope (Chang, 1998). The belief that one is capable of exercising control over one's functioning and coping is a central feature of self-efficacy theory (Bandura, 1997). Self-efficacy theory suggests that our perception of situational success, leads to increased perseverance toward producing desired outcomes. As a result, individuals with high self-efficacy adopt more adaptive, problem-focused, and less avoidance coping strategies (Luszczynska et al., 2005; Schwarzer et al., 2005). Perhaps not surprisingly, in an injury context, higher self- and treatment efficacy have been directly associated with adherence to rehabilitation programmes (Taylor and May, 1996; Brewer et al., 2003; Levy et al., 2008), and higher perceived self-efficacy of pre-surgery knee function has been found to be predictive of levels of physical activity, ongoing symptoms, and muscle function 1 year post ACL reconstruction (Thomeé et al., 2008).

Although Lazarus and Folkman (1984) consider control and self-efficacy appraisals to be similar, Bandura (1986) suggested that they are separate, with self-efficacy mediating the relationship between control appraisals and coping. Furthermore, despite sharing a substantial amount of conceptual overlap, optimism and self-efficacy are expectation beliefs at different levels of generality (Williams, 2010). While optimism represents a stable personality trait, self-efficacy involves situation-specific beliefs about ones capabilities to perform certain behaviors (Bandura, 1997). Therefore, it is likely that patients' efficacy beliefs will fluctuate during lengthy rehabilitation periods as a function of the stressors associated with the different stages of rehabilitation (Evans et al., 2012).

Unfortunately, despite its implications for clinical practice and recovery outcomes, the relationship between stable personal characteristics (e.g., personality traits), and more transitory cognitive, emotional, and behavioral responses across rehabilitation remains poorly understood (Brewer, 2010). Conceptual and methodological limitations have undoubtedly contributed to this. Conceptually, researchers have rarely accounted for the complex interplay between psychosocial factors, psychological responses and recovery outcomes. Instead, researchers have typically examined the direct relationships between psychosocial factors and psychological responses (e.g., Levy et al., 2008) or recovery outcomes (e.g., Thomeé et al., 2008) independently of each other. Methodologically, even fewer studies have adopted prospective repeated measures designs that capture the full rehabilitation period, which is essential when examining the mediating relationships between psychosocial factors, psychological responses and recovery outcomes.

By its very nature mediation implies changes over time, and any test of mediation with cross-sectional data can generate substantially biased (and thus potentially seriously misleading) estimates of longitudinal parameters (Maxwell and Cole, 2007). To the best of our knowledge, only six other studies within the psychology of sport injury literature have set out to examine mediation effects (Brewer et al., 2000; Levy et al., 2008; Chan et al., 2009; Wadey et al., 2012, 2013, 2014). Of these, two studies (Chan et al., 2009; Wadey et al., 2014) were based on a completely cross-sectional design, two studies (Wadey et al., 2012, 2013) overlooked the longitudinal structure of their own data by focusing on single waves in isolation (i.e., tested the independent, mediator, and dependent variables at the same time point—either onset, rehabilitation, or return-to-sport), one study (Levy et al., 2008) adopted a half-longitudinal design in which primary factors (independent variable) and intentions (mediator variable) were assessed at the same time, and one study (Brewer et al., 2000) didn't test for mediation because none of the adherence measures were significantly correlated with both psychosocial and outcome measures. While the findings from these studies have done much to advance our understanding of the relationships between variables of interest, the results should be interpreted with caution. Beyond this ability to eliminate bias in parameter estimates, longitudinal, repeated-measure designs can also yield important information about temporal precedence (MacKinnon et al., 2002), that is, which variables are causes and which are effects, which is so important when examining these conceptual relationships.

**Figure 1:**
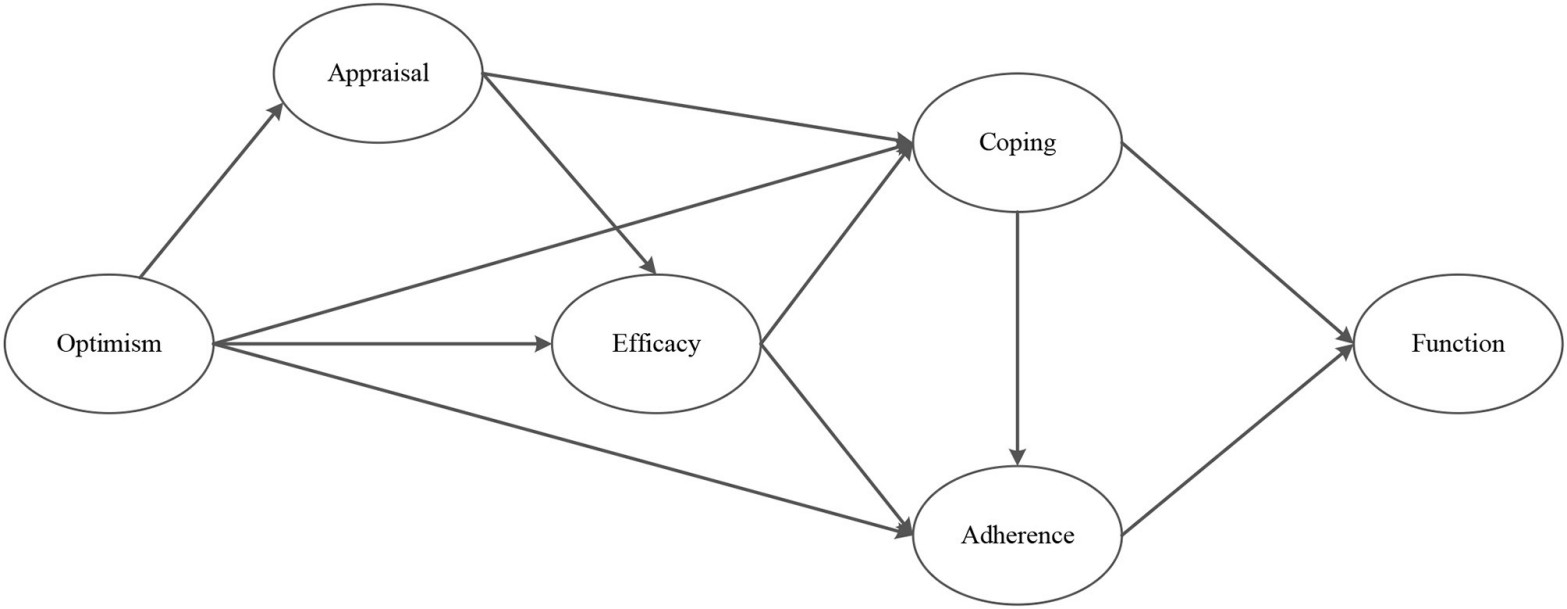
Hypothesized model of the relationship between optimism, psychosocial factors and perceived knee function following ACL surgery.

Therefore, the aim of this study was to address these shortcomings by examining the mechanisms through which optimism and psychosocial factors exert an effect on recovery outcomes throughout rehabilitation following ACL reconstruction. To achieve this aim, we tested the mediated effects depicted in Figure 1 throughout the first year of recovery following ACL knee reconstruction. Based on the integrated model of psychological response to injury (Wiese-Bjornstal et al., 1998), the biopsychosocial model of injury rehabilitation (Brewer et al., 2002), stress and coping theory (Lazarus and Folkman, 1984), and Bandura's (1986, 1997) self-efficacy theory, the following hypotheses were proposed:

- Optimism will have an indirect effect on perceived knee function, via rehabilitation adherence (Hypothesis 1), and coping (Hypothesis 2);
- Coping will have an indirect effect on perceived knee function, via rehabilitation adherence (Hypothesis 3);
- Optimism will have an indirect effect on rehabilitation adherence, via efficacy (Hypothesis 4);
- Optimism will have an indirect effect on coping, via cognitive appraisal (Hypothesis 5) and efficacy (Hypothesis 6);
- Optimism will have an indirect effect on efficacy, via cognitive appraisal (Hypothesis 7).

## Methods

### Participants

An initial pre-surgery sample of 190 patients were recruited for the study based on a number of criteria. Specifically, participants were required to: (a) be undergoing primary ACL reconstruction with one of three orthopedic surgeons' associated with this study; (b) be 18 years of age or older; and (c) have sustained their ACL injury through participation in sport (i.e., training or competition). Patients undergoing revision surgery, and/or additional procedures other than simple meniscal/chondral surgery were subsequently excluded from the study to minimize the error variance associated with their type of injury, surgical technique, and prognosis. Participants who failed to complete all study measures at all time-points were also excluded. As a result, the final sample comprised 81 injured participants (51 males and 30 females) who ranged in age from 18 to 55 years old (*M* = 26.9 years; *SD* = 7.5), and represented team (77%) and individual (23%) sports, at national (12%), regional (40%), club (33%), and recreational (15%) level.

### Measures

#### Optimism

The Life Orientation Test-Revised (LOT-R; Scheier et al., 1994) assessed dispositional optimism. The LOT-R is a 10-item measure consisting of six-scale items (three items worded in a positive direction and three in a negative direction), and four filler items. Participants rated the extent of their agreement with each item (e.g., “Overall, I expect more good things to happen to me than bad”) on a 5-point Likert scale anchored at 0 (*strongly disagree*) and 4 (*strongly agree*). Negatively worded items were reversed, and the sum of the items yielded an optimism score ranging from 0 to 24. Previous research has reported internal reliability estimates (Cronbach's alpha) of 0.78 for the LOT-R (Scheier et al., 1994). Despite extensive use across a variety of medical contexts (e.g., Giltay et al., 2004; Tindle et al., 2012; Kim et al., 2017), there has been some debate about whether the optimism construct exists as a bipolar dimension, or whether there are two separate dimensions—one pertaining to the affirmation of optimism and one pertaining to the affirmation of pessimism (Scheier and Carver, 2018). In light of these concerns, we conducted confirmatory factor analysis (CFA) on the LOT-R to assess its factorial validity before proceeding with the main analysis. Cronbach's alpha coefficients for the present study ranged from 0.77 to 0.83 across time points.

#### Coping Strategies

The Coping with Health Injuries and Problems (CHIP; Endler et al., 1998) assessed pre- and post-surgery coping strategies. The CHIP is a 32 item measure consisting of four subscales: instrumental coping (e.g., “Find out more information about my injury”), emotional preoccupation (e.g., “Feel anxious about being weak and vulnerable”), distraction coping (e.g., “Daydream about pleasant things”), and palliative coping (e.g., “Lie down when I feel tired”). Participants indicated the extent to which they engaged in each coping strategy on a 5-point Likert scale (1 = *not at all*; 5 = *very much*). Total scores for each subscale range from 8 to 40. The CHIP was developed for use with populations experiencing physical and health related problems as opposed to sports injuries specifically. While population-specific measures are always the preferred approach, in the absence of this, particularly when the CHIP has been used so widely across a number of domains including sports injury (e.g., Udry, 1997; Carson and Polman, 2010), we deemed it to be the most appropriate coping measure for this study. Cronbach's alpha coefficient for distraction (0.75 to 0.80), palliative (0.71 to 0.80), instrumental (0.75 to 0.84), and emotion (0.85 to 0.90) subscales were reported in the present study.

#### Rehabilitation Beliefs

The Sport Injury Rehabilitation Beliefs Survey (SIRBS; Taylor and May, 1996) assessed participants' perceptions about their injury and post-surgery rehabilitation. SIRBS is a 19-item measure comprising five subscales; severity (*n*_items_ = 5; e.g., “This injury is too serious to not follow medical advice”), susceptibility (*n*_items_ = 5; e.g., “My recovery from injury may be hindered if I do not complete the rehabilitation programme”), treatment efficacy (*n*_items_ = 4; e.g., “I have absolute faith in the effectiveness of my rehabilitation programme”), self-efficacy (*n*_items_ = 4; e.g., “I will have no serious difficulties in following the instructions of my rehabilitation programme”), and value of rehabilitation (*n*_items_ = 1; “Being fully recovered from injury is extremely important to me”). Items are rated on a 7-point Likert scale anchored at *very strongly disagree* (1) and *very strongly agree* (7). Taylor and May (1996) reported alpha coefficients of 0.52, 0.84, 0.85, and 0.91 for the severity, susceptibility, treatment efficacy, and self-efficacy subscales, respectively. Cronbach's alpha coefficients in the present study were similar, ranging from 0.59 to 0.74, (severity) 0.81 to 0.86 (susceptibility), 0.80 to 0.84 (treatment efficacy), and 0.85 to 0.90 (self-efficacy).

#### Appraisals

Two measures of appraisals were included in this study. Firstly, appraisals were examined using six separate items used by Chang (1998). Participants responded on a 5-point Likert scale ranging from 1 (*not at all*) to 5 (*very much so*) to four items assessing primary appraisal (“How important is your full recovery?,” “How threatening [could have negative consequences for you] do you find your rehab?,” “How challenging [could have positive consequences for you] do you find your rehab?,” “How much stress does your rehab cause you?”), and two items assessing secondary appraisals (“How much control do you feel you have over the outcome?,” “How effectively do you feel you are able to cope with your rehab?”). Secondly, the 24-item Sport-Injury Appraisal Inventory (SIAI; unpublished: Waters, 2005) explored aspects of confidence specific to injury rehabilitation, fear of re-injury, and impact of injury on self. The inventory comprises five subscales: confidence in own body (*n*_*items*_ = 4; e.g., “I cannot trust my body anymore”), confidence in rehabilitation team (*n*_*items*_ = 4; e.g., “I have confidence in the professional ability of my physiotherapist”), confidence about ability on re-entry into sport (*n*_*items*_ = 4; “Right now, I feel apprehensive about my ability to perform in competition”), impact of injury on self (*n*_*items*_ = 7; e.g., “I feel that I am never going to be the same person that I was prior to my injury”), and fear of re-injury (*n*_*items*_ = 5; e.g., “I worry that my injured body part will not hold up in a competition”). Participants responded on a 7-point Likert scale anchored at 1 (*very strongly disagree*) and 7 (*very strongly agree*). Cronbach's alpha coefficient for primary appraisal (0.80 to 0.86), secondary appraisal (0.81 to 0.89), confidence in own body (0.67 to 0.85), confidence in rehabilitation team (0.86 to 0.91), confidence about ability on re-entry into sport (0.74 to 0.84), impact of injury on self (0.79 to 0.86), and fear of re-injury (0.84 to 0.91) were reported in this study.

#### Adherence

Two post-surgery measures were used to assess rehabilitation adherence. Firstly, and consistent with previous research (e.g., Levy et al., 2008), an attendance ratio was calculated for each participant from the number of rehabilitation appointments they attended compared to the number of appointments recommended by their physiotherapist. For example, if participants attended 12 out of 24 scheduled appointments their adherence ratio would be 0.50. Thus, adherence ratios could range from 0 to 1.0. Secondly, we adopted the same three separate items of home-based rehabilitation as a number of previous studies (e.g., Brewer et al., 2000; Bassett, 2003; Levy et al., 2008). Participants responded on a 5-point Likert scale ranging from 1 (*not at all)* to 5 (*as advised*) as to the extent to which they had (a) completed the recommended home exercises, (b) refrained from undertaking activity that could harm the injury and (c) applied home cryotherapy (icing). The sum of the three items represented a total score for home rehabilitation adherence at each time point, with the total ranging from 3 to 15. Cronbach's alphas for home rehabilitation in this study were 0.67 (1 month), 0.71 (2 months), 0.79 (3 months), and 0.80 (6 months).

#### Perceived Knee Function

The 18-item International Knee Documentation Committee Subjective Knee Form (IKDC; Irrgang et al., 2001) assessed perceptions of pain, stiffness, swelling, joint locking, and joint instability. In its original form, response types include 5-point Likert scales, 11-point Likert scales, and dichotomous “yes-no” responses, which are calculated to form a total perceived knee function score. However, given concerns about the factorial integrity of IKDC (cf. Grevnerts et al., 2015; Williams et al., 2020), a validated two-factor conceptualization proposed by Higgins et al. (2007), which reflected symptoms and knee articulation (SKA; *n*_items_ = 11), and activity level (AL; *n*_items_ = 4), was employed in this study. Cronbach's alpha coefficients for these subscales across time points ranged from 0.71 to 0.91 in this study.

### Procedure

Following University's Research Ethics Committee ethical approval, the consulting surgeons identified suitable participants based on the inclusion/exclusion criteria, explained to them the nature of the study, and invited them to participate at their pre-operation consultation. Thereafter, participants completed a pre-surgery booklet comprising informed consent, demographic information, the LOT-R, CHIP, and IKDC. Post-surgery patient details were given to the researcher to collect the post-surgery surveys, which were sent to each participant electronically via a “Survey Monkey” email link at 1, 2, 3, 6, and 12 months post-surgery. Post-surgery surveys comprised the LOT-R, CHIP, SIRBS, SIAI, appraisal items, and an assessment of home-based rehabilitation. Participants completed the post-surgery measures at 1, 2, 3, 6, and 12 months. The IKDC was included in the electronic surveys at 6 and 12 months to assess perceived knee function. The physiotherapist prospectively recorded attendance at rehabilitation appointments, which was then sent to the first author at the end of formalized rehabilitation.

### Data Analysis

Of an initial 190 participants that completed the pre-surgery measures, a total of 81 participants completed all measures across the six time points. Missing data at an item-level in the pre-surgery measures (administered in hard copy), were treated using mean imputation (Kline, 2015). Data analysis involved four stages. First, we conducted CFA to assess whether dispositional optimism should be examined as one-factor (optimism) or two-factors (optimism and pessimism) for the remainder of the analysis. Second, one-way between groups multivariate analysis of variance (MANOVAs) were performed before undertaking the mediation analysis to determine the potential confounding effects of the demographic variables, age, gender, competitive level, and operating surgeon on the two measures of adherence and perceived knee function (SKA and AL). Third, this was an exploratory study of complex relationships that had not been examined collectively. The original model with all study variables (i.e., primary and secondary appraisals, all four coping subscales, home rehabilitation and rehabilitation adherence ratio), was therefore, too complex (relative to our sample size) to test in its entirety, so we simplified it by testing partial mediation models (i.e., Hypotheses 1–7) first. Finally, we incorporated the findings from this third stage of the analysis to revise and test the simplified conceptual model that appears in Figure 11. Bayesian structural equation modeling (BSEM; Muthén and Asparouhov, 2012) with Mplus version 8 was used to examine the factorial validity of the LOT-R (stage one of the analysis) and the indirect relationships between optimism, psychosocial factors, rehabilitation adherence, and perceived knee function (stages three and four of the analysis).

Bayesian estimation was employed in this study because it offers a more flexible analytic approach to overcome the highly restrictive features commonly applied within CFA, in which indicators are free to load on their intended factors, but cross-loadings and residual correlations are fixed at zero. This can be argued to be a strongly simplified representation of real measurement situations, which almost always leads to the rejection of the model by the likelihood ratio χ^2^ test (Marsh et al., 2009). Instead, BSEM enables researchers to model uncertainty in their specifications by replacing exact zero parameters with approximate zeros (i.e., zero mean, small variance; Muthén and Asparouhov, 2012). In doing so, indicators have a major loading on a hypothesized factor but small cross-loadings are possible due to a minor influence from other factors, better reflecting substantive theories (Muthén and Asparouhov, 2012).

In addition to BSEM being a more flexible analytic approach to CFA, several recent methodological papers and simulation studies have also reported the apparent advantages of Bayesian methods over more traditional approaches with small sample sizes (e.g., Depaoli, 2013; McNeish, 2016; Depaoli and van de Schoot, 2017). Due to different theoretical underpinnings (see Gelman et al., 2004), Bayesian methods do not require large sample sizes. Sampling-based Bayesian methods such as the Markov Chain Monte Carlo (MCMC) ensure that the quality of the inference is controlled by the number of samples taken approaching infinity, rather than the sample size approaching infinity (Lee and Song, 2004; Kruschke, 2010). In essence, MCMC models are better equipped to model small-sample data because they don't rely on asymptotics in the same way as frequentist methods (Muthén and Asparouhov, 2012; Depaoli and van de Schoot, 2017; van de Schoot et al., 2017). However, while Bayesian methods are better suited to model data with small sample sizes, the estimates are highly sensitive to the specification of the prior distribution (McNeish, 2016).

#### Model Specification

##### CFA on the LOT-R

To assess whether dispositional optimism should be treated as one (optimism) or two (optimism and pessimism) separate factors a series of BSEMs were estimated. First, the estimation of the one-factor and two-factor models incorporated non-informative priors for the major loadings, exact zero cross-loadings and exact zero residual correlations. Second, the estimation of the two-factor model incorporated the addition of informative approximate zero cross-loadings. The estimation of the final models incorporated informative approximate zero residual correlations for the one-factor model, and both informative approximate zero cross-loadings and residual correlations for the two-factor model. We specified small prior variances for cross loadings with a mean of zero and a variance of 0.01, corresponding to 95% small cross-loading bounds of ±0.02 based upon statistical recommendations (e.g., Muthén and Asparouhov, 2012). For the correlated residuals we specified an inverse-Wishart prior distribution IW (0, degrees-of-freedom parameter d = p + 6). Finally, to examine the influence of the specification of different prior variances on the posterior predictive *p-*value (PPP) and the stability of the estimates (Muthén and Asparouhov, 2012), we performed a sensitivity analysis by comparing estimates with priors on the cross-loadings specified at 0.015, 0.01, and 0.005, and priors on the residual correlations specified at IW 0, *d* = p + 6, p + 20, and p + 2 to check for any important discrepancies.

##### Bayesian Mediation Analysis

During stage three of the analysis we standardized the variables (optimism, psychosocial factors, rehabilitation adherence, perceived knee function) and modeled the hypothesized structural paths (i.e., Hypotheses 1-7) using simple mediated models (e.g., time 1 optimism; time 2 rehabilitation adherence; time 3 knee function) as opposed to more complex mediation models such as autoregressive (e.g., cross-lagged mediation) models because, despite BSEMs better small sample performance (e.g., Stenling et al., 2015), the full cross-lagged model with all possible structural paths was too complex for the current sample size. Simple mediated effects occur when the predictor variable (e.g., optimism) changes a mediator (e.g., rehabilitation adherence; the α path) and the mediator changes the outcome variable (e.g., perceived knee function; the β path). The mediated effect is then the product of α and β paths (αβ). Similar to the conventional standard error, the posterior standard deviation (post. *SD*) provides a Bayesian measure of estimation uncertainty. However, unlike frequentist mediation analysis that utilize confidence intervals, Bayesian estimation uses a 95% credibility interval to test the significance levels of the indirect effects. Indirect effects are significant if their 95% credibility interval does not include zero (Yuan and MacKinnon, 2009).

All constructs within the mediation models, except the adherence ratio, were modeled as latent variables. We used the subscales scores from the IKDC (SKA and AL) to create the latent factor, “knee function,” and the subscales from the SIRBS (self- and treatment efficacy) and SIAI (confidence in own body, confidence in rehabilitation team and confidence about ability on re-entry into sport) to create the latent factor, “efficacy.” “Primary appraisal,” “secondary appraisal,” and “home rehabilitation” latent variables were indicted by their original items. Finally, the latent variables, “optimism” and each of the separate coping dimensions (e.g., “instrumental coping,” “emotion coping,” “distraction coping,” and “palliative coping”) were indicated by three and two parcels respectively. Each parcel was constructed following a factorial algorithm parceling approach (cf. Matsunaga, 2008). A factor analysis was conducted on each measure (e.g., 6 items of the LOT-R), with the parcels then created based on the magnitude of the loadings. This approach was employed to avoid specifying latent variables with only one indicator, which causes under-identified (i.e., df <0) models that cannot be computed (Kline, 2015). Once more we specified small prior variances for cross loadings with a mean of zero and a variance of 0.01, and an inverse-Wishart prior distribution IW (0, degrees-of-freedom parameter d = p + 6) for the correlated residuals. We conducted the same sensitivity analysis as the CFA for a randomly selected number of simple mediation models (stage three) and the conceptual model (stage four).

#### Model Testing

Model convergence was examined using the MCMC simulation procedure with a Gibbs sampler (Asparouhov and Muthén, 2010; Muthén and Asparouhov, 2012), a fixed number of 100,000 iterations, and the potential scale reduction factor (PSR), where evidence for convergence is demonstrated when the PSR lies between 1.0 and 1.1 for all parameters (Gelman et al., 2004). The PPP value and 95% confidence interval for the difference in the observed and replicated χ^2^ values is used to assess model fit. A good fitting model is indicated when the PPP values are around 0.50 (Muthén and Asparouhov, 2012), and the 95% confidence interval values center on zero (Muthén and Asparouhov, 2012). Furthermore, the Deviance Information Criterion (DIC) value is also used to compare measurement invariance models in the Bayesian estimation, in which a lower value indicates a better fitting model (Asparouhov et al., 2015).

## Results

### CFA on the LOT-R

Before undergoing ACL reconstructive surgery, 190 patients completed the 10-item LOT-R. Table 1 provides an overview of the model fit statistics for the one and two-factor optimism models with this initial sample. Both the one and two-factor models demonstrated significantly better fit statistics with the inclusion of informative priors on the residual correlations (one-factor) and both the cross-loadings and the residual correlations (two-factor). The one-factor model (PPP = 0.519, Δobserved and replicated χ^2^ 95% CI [-20.99, 20.38]) indicated slightly better fit (i.e., greater PPP value) than the two-factor model. Furthermore, the sensitivity analysis revealed no important discrepancies between parameter estimates when varying the a priori distribution for cross-loadings and residual covariance. Subsequently, and in accordance with other researchers (e.g., Carver et al., 2010; Monzani et al., 2014), dispositional optimism was treated as one bipolar dimension for the remainder of the analysis.

**Table 1:** BSEM Fit for the Life Orientation Test-Revised Scale.

**Model**	**DIC**	**PPC 95% CI**	**PPP value**
**One-factor**			
Non-informative Priors	4628.429	11.513 – 49.753	0.001
Informative Priors (residual correlations)	4605.647	−20.988 – 20.379	0.519
**Two-factor**			
Non-informative Priors	4631.53	13.25 – 53.31	0.001
Informative Priors (cross-loadings)	4612.36	−9.53 – 38.09	0.130
Informative Priors (cross-loadings + residual correlations)	4604.190	−20.844 – 20.311	0.514

### Descriptive Statistics

Table 2 contains the descriptive statistics for the study variables at each time point. One-way MANOVAs revealed no statistically significant differences between adherence and perceived knee function at pre, 6, or 12 months post-operation for age, competitive level, and operating surgeon. There was, however, a statistically significant difference for gender [*F*_(2, 57)_ = 6.71, *p* = 0.002; Wilks' Lambda = 0.80]. When the dependent variables were considered separately using a Bonferroni adjusted alpha level of 0.025, only perceived knee function reached statistical significance [*F*_(1, 58)_ = 11.23, *p* = 0.001, partial eta squared = 0.17]. An inspection of the mean scores indicated males (*M* = 59.67, *SD* = 14.13) reported significantly higher perceived knee function prior to surgery compared to females (*M* = 46.96, *SD* = 13.64). Consequently, gender was controlled in all subsequent analyses.

**Table 2:** Means Scores with Standard Deviation (in parentheses).

**Measure**	**Pre-surgery**	**1 Month**	**2 Months**	**3 Months**	**6 Months**	**12 Months**
Optimism	15.36 (3.55)	14.60 (3.44)	15.11 (3.63)	15.12 (3.70)	15.52 (3.58)	-
Distraction coping	26.31 (5.32)	25.09 (5.07)	25.20 (3.97)	25.11 (4.41)	24.67 (4.91)	-
Palliative coping	20.19 (4.00)	21.36 (4.71)	20.48 (3.94)	18.93 (4.13)	15.80 (3.39)	-
Instrumental coping	30.13 (5.03)	29.37 (5.12)	28.69 (4.68)	28.04 (5.57)	26.40 (5.80)	-
Emotion coping	23.20 (7.82)	23.64 (7.57)	23.11 (7.69)	22.73 (7.72)	21.85 (6.78)	-
Treatment efficacy	-	20.96 (4.06)	20.71 (3.81)	20.44 (3.96)	-	-
Self-efficacy	-	21.16 (4.66)	20.52 (4.35)	20.52 (4.56)	-	-
Confidence in own body	-	16.07 (5.15)	16.20 (4.84)	16.49 (4.70)	-	-
Confidence in rehab team	-	21.01 (4.95)	20.71 (4.28)	21.93 (4.06)	-	-
Confidence in ability to re-enter	-	13.21 (5.16)	13.25 (5.28)	12.93 (4.99)	-	-
Primary appraisal	-	13.63 (2.57)	13.71 (2.25)	13.61 (1.93)	-	-
Secondary appraisal	-	6.80 (2.03)	6.85 (1.86)	7.03 (1.90)	-	-
Home rehabilitation	-	12.08 (2.17)	11.39 (2.30)	10.79 (2.18)	9.85 (2.35)	-
Rehabilitation adherence ratio					0.81 (0.14)	-
Symptoms and knee articulation	-	-	-	-	-	4.52 (0.54)
Activity level	-	-	-	-	-	2.98 (0.50)

### Bayesian Mediation Analysis

All of the following mediation models demonstrated excellent model fit statistics based on appropriate PPP values, observed and replicated χ^2^ 95% CI, and model convergence as verified by visual inspection of trace plots and PSR development over time (cf. Gelman et al., 2004; Muthén and Asparouhov, 2012). Furthermore, all intended factor loadings of the latent variables from the observed variables were good (>0.55) and significant, with all cross-loadings small (< ±0.15) and non-significant.

#### Hypothesis 1

**Figure 2:**
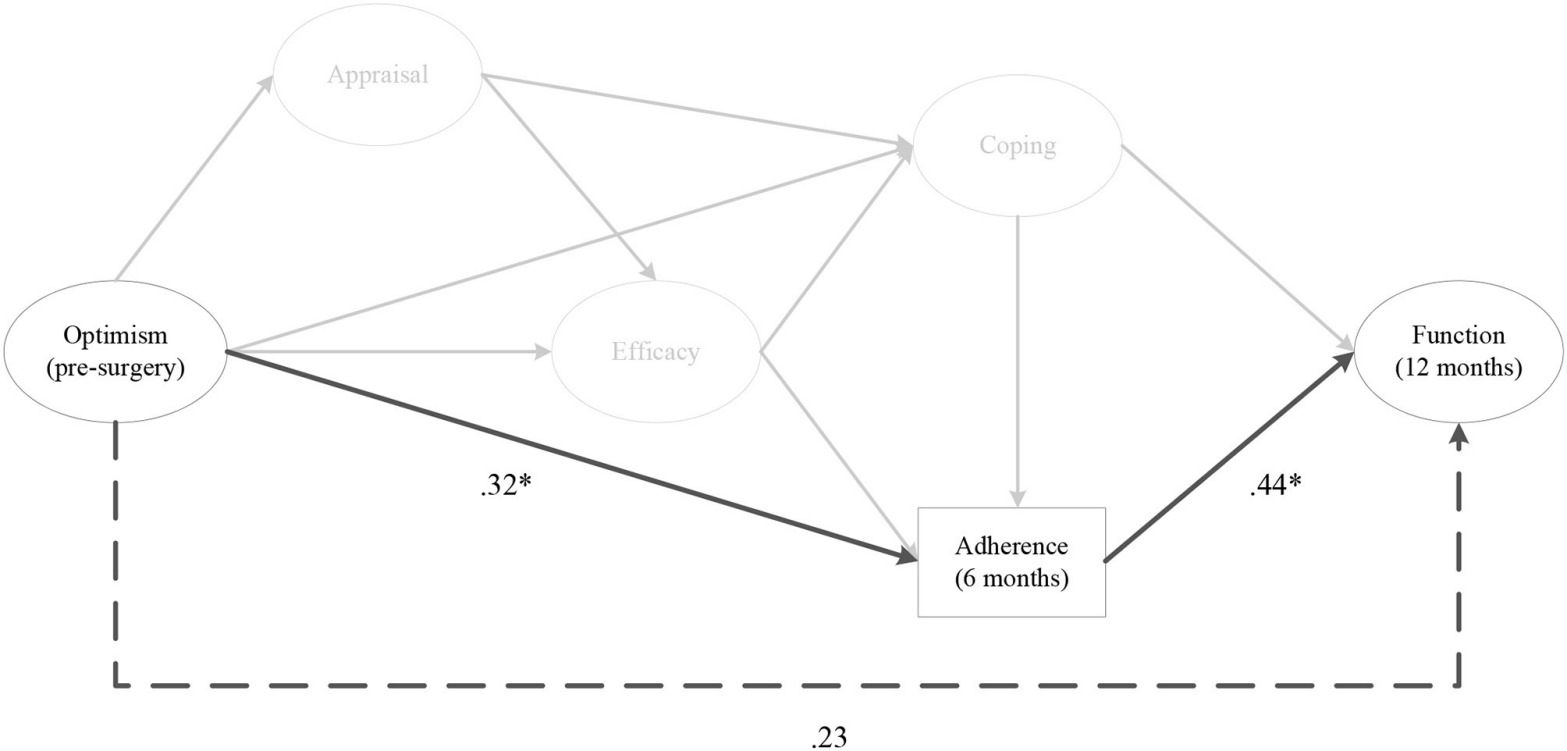
The indirect effect of pre-surgery optimism on perceived knee function at 12 months post-surgery, via rehabilitation adherence ratio at 6 months post-surgery. *Note*. Parameter estimates are standardized; dashed lines indicate non-significant paths. *p < 0.05.

As depicted in Figure 2, the indirect effects of pre-surgery optimism on 12 months post-surgery perceived knee function, via rehabilitation adherence ratio at 6 months post-surgery was significant (αβ = 0.13, post. *SD* = 0.07, CI [0.02, 0.31]). The same relationship existed when the model was run with optimism at 1, 2, and 3 months post-surgery. There were no indirect effects for optimism on perceived knee function, via home rehabilitation adherence at any time points.

#### Hypothesis 2

**Figure 3:**
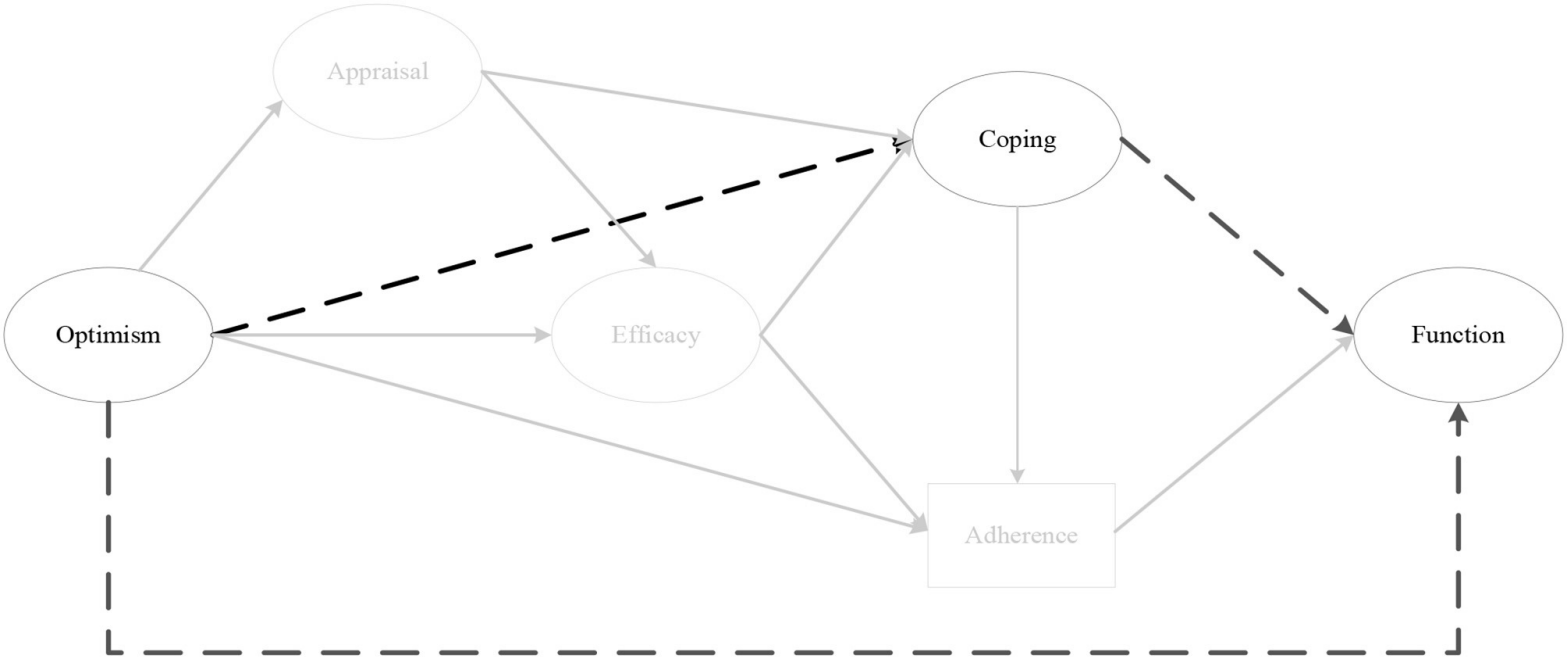
The indirect effect of optimism on perceived knee function, via coping. *Note*. Parameter estimates are standardized; dashed lines indicate non-significant paths.

There were no indirect effects for optimism on perceived knee function, via any coping strategies at any time points (Figure 3). However, there were consistent negative direct effects for emotion coping. Specifically, pre-surgery optimism was negatively associated with emotion coping at 1 month (*a* = −0.50, CI = [−0.94, −0.12]), 2 months, 3 months, and 6 months post-surgery. Optimism at 1 month post-surgery was negatively associated with emotion coping at 2 months (*a* = −0.45, CI = [−0.69, −0.11]), 3 months, and 6 months post-surgery. Optimism at 2 months post-surgery was negatively associated with emotion coping at 3 months (*a* = −0.43, CI = [−0.68, −0.11]) and 6 months post-surgery. Finally, optimism at 3 months post-surgery was negatively associated with emotion coping at 6 months (*a* = −0.40, CI = [−0.64, −0.04]) post-surgery. No other direct effects for coping were observed.

#### Hypothesis 3

**Figure 4:**
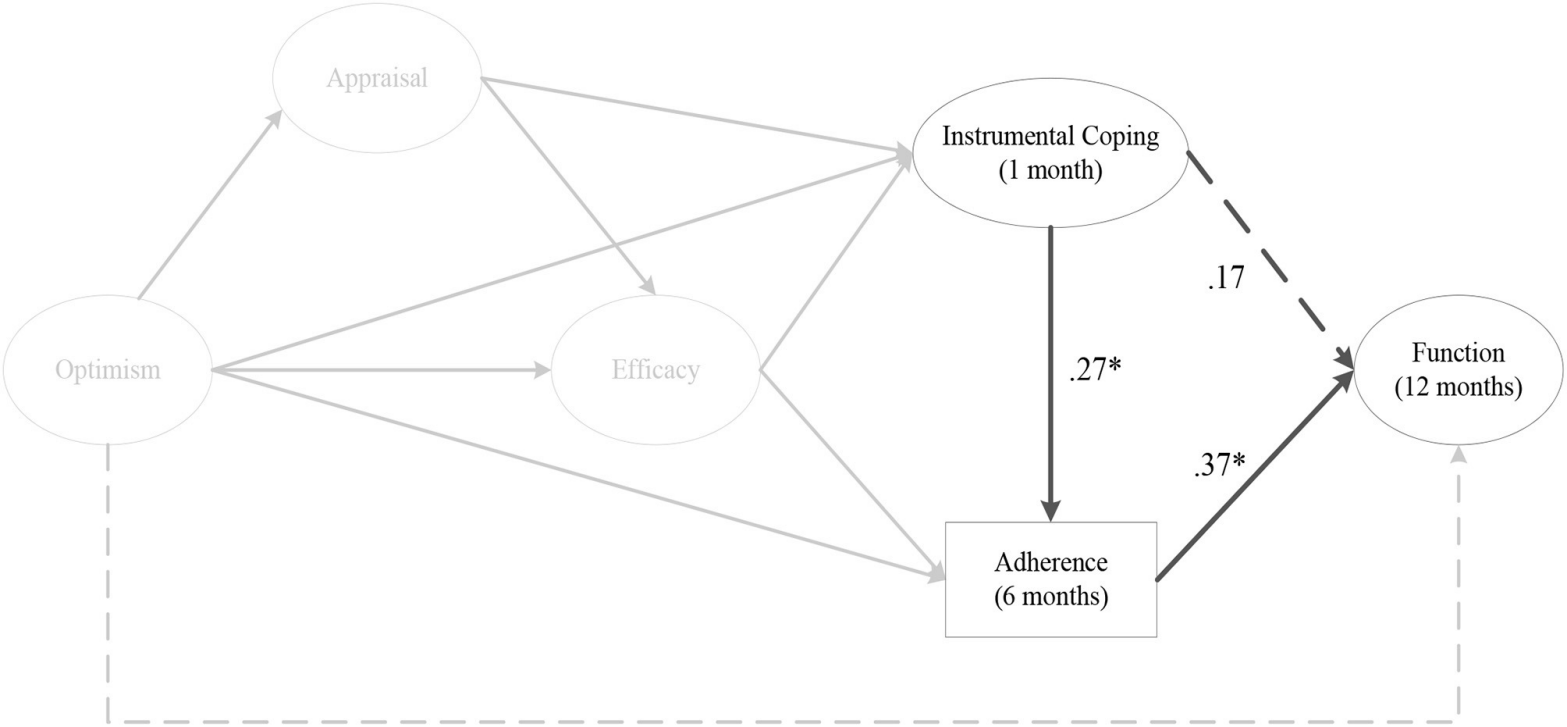
The indirect effect of instrumental coping at 1 month post-surgery on perceived knee function at 12 months post-surgery, via rehabilitation adherence ratio at 6 months post-surgery. *Note*. Parameter estimates are standardized; dashed lines indicate non-significant paths. *p < 0.05.

**Figure 5:**
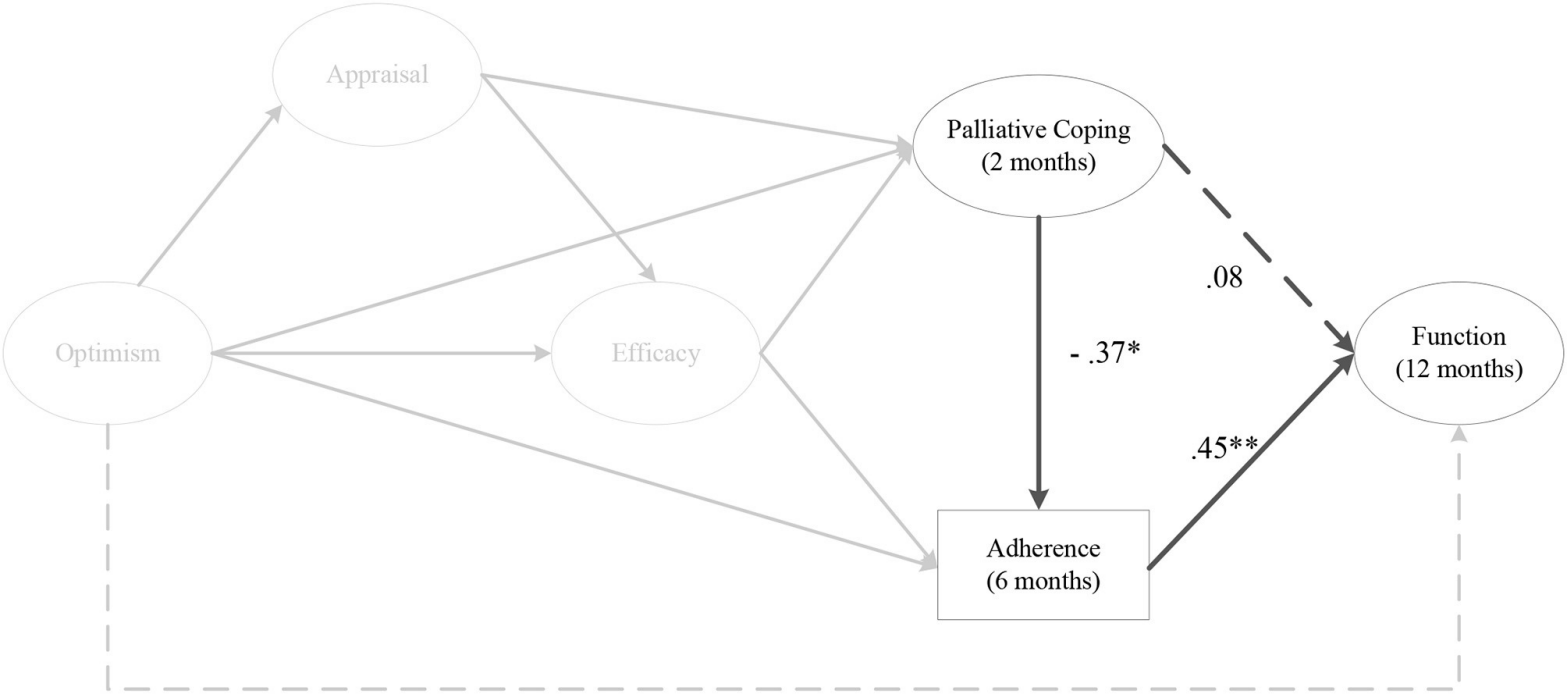
The indirect effect of palliative coping at 2 months post-surgery on perceived knee function at 12 months post-surgery, via rehabilitation adherence ratio at 6 months post-surgery. *Note*. Parameter estimates are standardized; dashed lines indicate non-significant paths. *p < 0.05, **p < 0.01.

**Figure 6:**
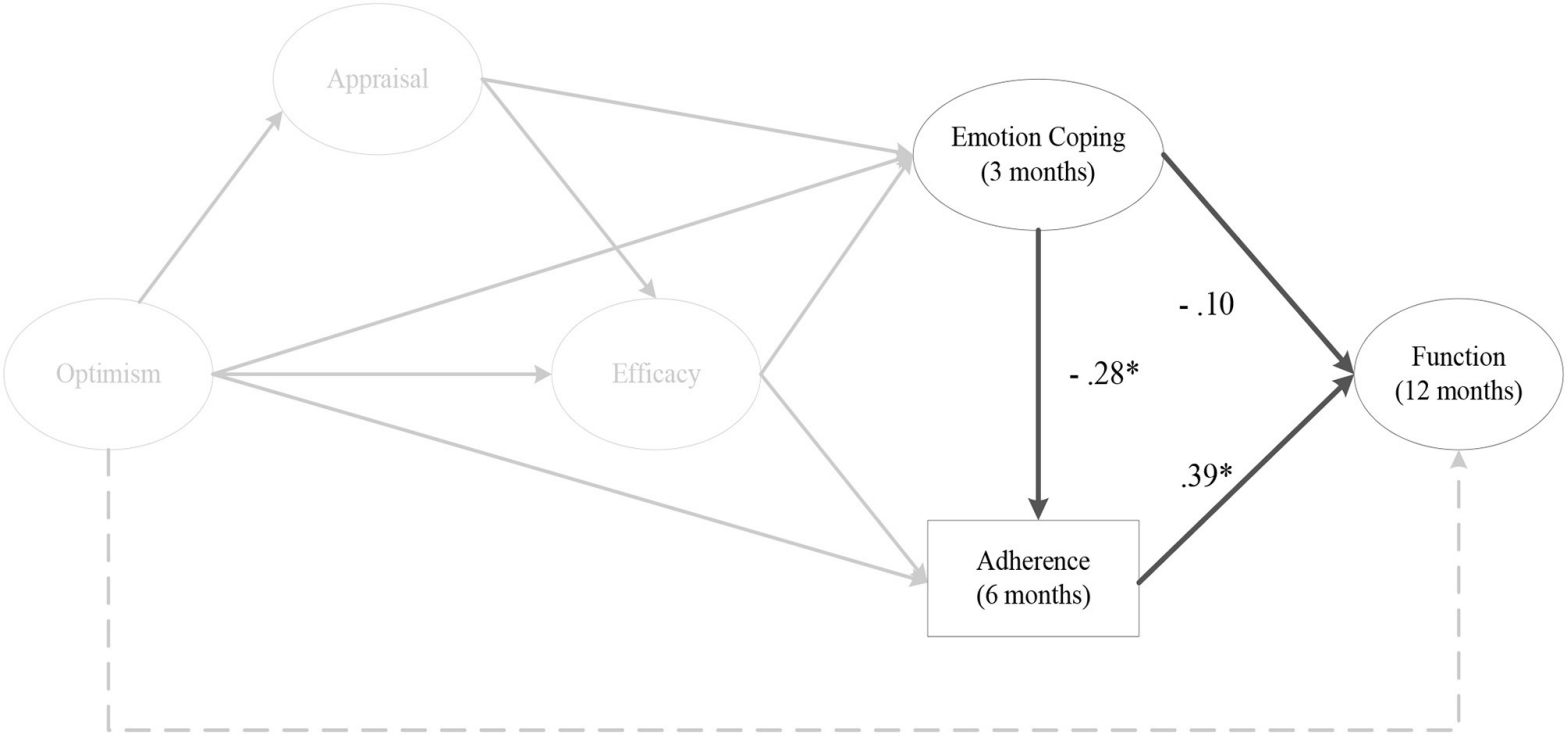
The indirect effect of emotion coping at 3 months post-surgery on perceived knee function at 12 months post-surgery, via rehabilitation adherence ratio at 6 months post-surgery. *Note*. Parameter estimates are standardized; dashed lines indicate non-significant paths. *p < 0.05.

The indirect effect models for instrumental coping at 1 month (αβ = 0.11, post. *SD* = 0.06, CI [0.01, 0.26]) (Figure 4), and 2 months post-surgery on perceived knee function at 12 months post-surgery were fully mediated by rehabilitation adherence ratio at 6 months post-surgery. As depicted in Figure 5, there was a significant negative mediating effect for palliative coping at 2 months (αβ = −0.18, post. *SD* = 0.08, CI [−0.37, −0.05]) and 3 months post-surgery on perceived knee function at 12 months post-surgery, via rehabilitation adherence ratio at 6 months post-surgery. Moreover, emotion coping at 3 months post-surgery (αβ = −0.11, post. *SD* = 0.07, CI [−0.27, 0.01]) (Figure 6), displayed a negative mediated relationship on perceived knee function at 12 months post-surgery, via rehabilitation adherence ratio at 6 months post-surgery. There were no indirect effects for any coping strategies on perceived knee function, via home rehabilitation adherence at any time points.

#### Hypothesis 4

**Figure 7:**
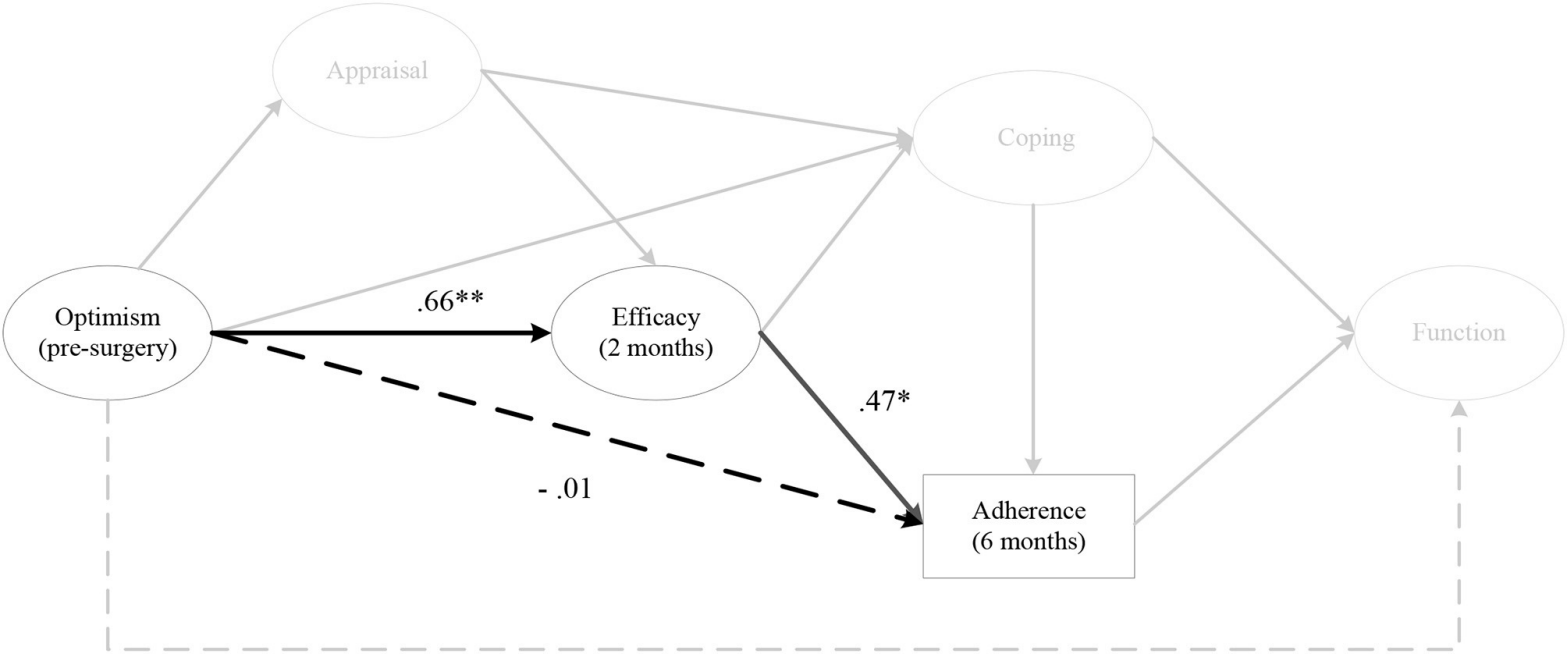
The indirect effect of pre-surgery optimism on rehabilitation adherence ratio at 6 months post-surgery, via efficacy at 2 months post-surgery. *Note*. Parameter estimates are standardized; dashed lines indicate non-significant paths. *p < 0.05, **p < 0.01.

The indirect effects of pre-surgery optimism on rehabilitation adherence at 6 months post-surgery, via efficacy at 2 months (αβ = 0.30, post. *SD* = 0.12, CI [0.11, 0.58]) (Figure 7) and 3 months post-surgery was significant. Furthermore, optimism at 1 month post-surgery on rehabilitation adherence at 6 months post-surgery, via efficacy at 2 months and 3 months post-surgery was significant, as was optimism at 2 months post-surgery on rehabilitation adherence at 6 months post-surgery, via efficacy at 3 months post-surgery. There were no indirect effects for optimism on home rehabilitation adherence, via efficacy at any time points.

#### Hypotheses 5 and 6

**Figure 8:**
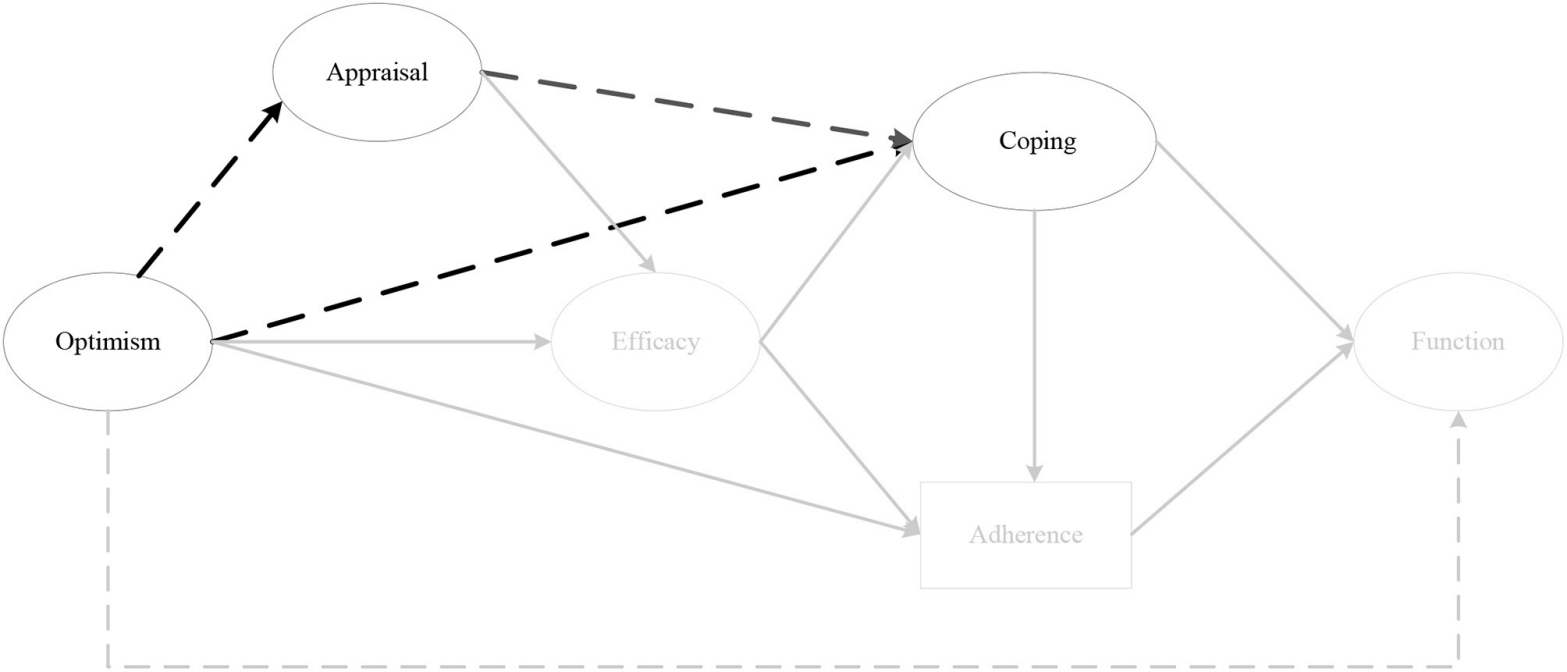
The indirect effect of optimism on coping, via cognitive appraisal. *Note*. Parameter estimates are standardized; dashed lines indicate non-significant paths.

There were no indirect effects for optimism on any of the coping strategies, via cognitive appraisal at any time points (Hypothesis 5) (Figure 8). However, there were a number of direct effects. Pre-surgery optimism was negatively associated with primary appraisal at 2 months (*a* = −0.46, CI = [−0.72, −0.08]), and 3 months post-surgery. Optimism at 1 month post-surgery was negatively associated with primary appraisal at 2 months (*a* = −0.44, CI = [−0.70, −0.08]), and 3 months post-surgery. Optimism at 2 months post-surgery was negatively associated with primary appraisal at 3 months post-surgery (*a* = −0.40, CI = [−0.67, −0.02]). Finally, primary appraisal at 3 months post-surgery was positively associated with emotion coping at 6 months post-surgery (*a* = 0.41, CI = [0.03, 0.73]). As for secondary appraisals, pre-surgery optimism was positively associated with secondary appraisal at 1 month (*a* = 0.54, CI = [0.19, 0.77]), 2 months, and 3 months post-surgery. Optimism at 1 month post-surgery was positively associated with secondary appraisal at 2 months (*a* = 0.66, CI = [0.38, 0.83]), and 3 months post-surgery. Furthermore, optimism at 2 months post-surgery was positively associated with secondary appraisal at 3 months post-surgery (*a* = 0.64, CI = [0.35, 0.83]).

**Figure 9:**
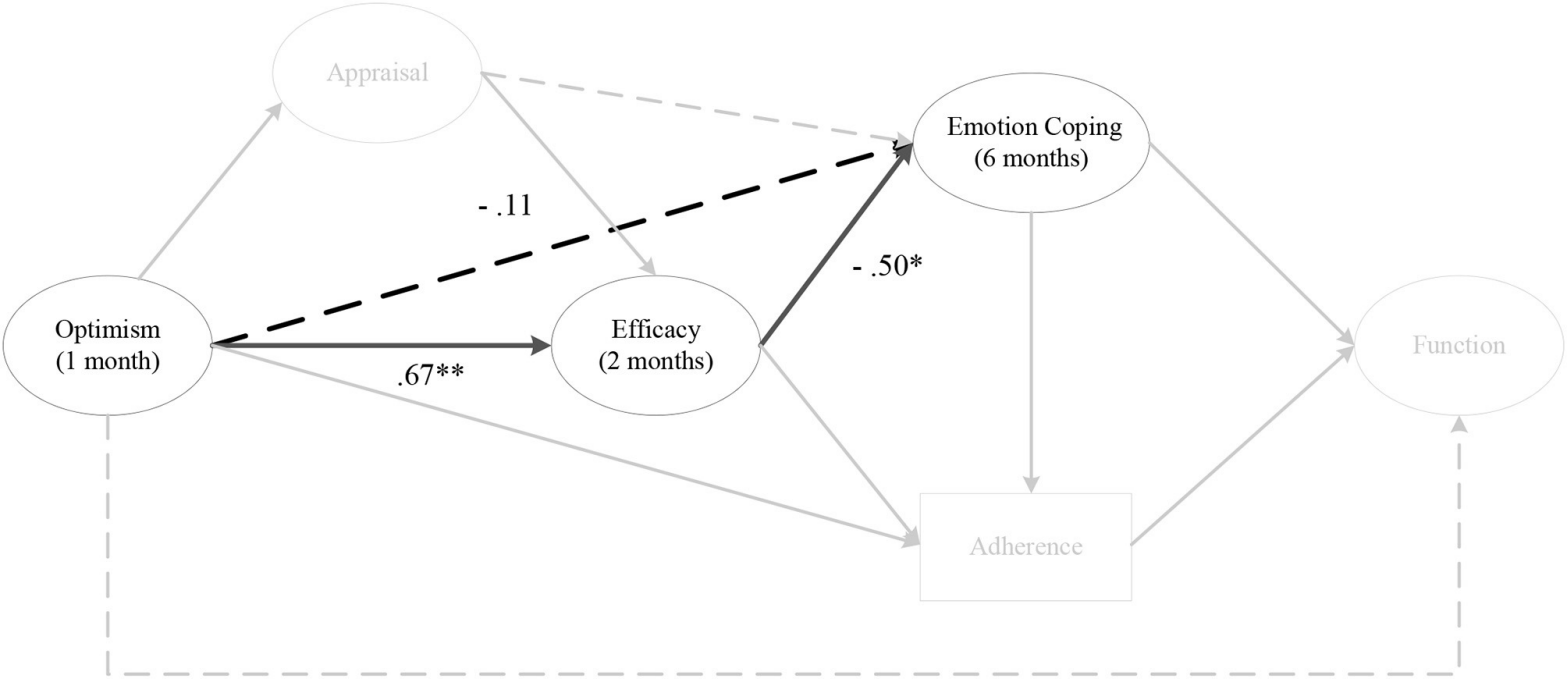
The indirect effect of optimism at 1 month post-surgery on emotion coping at 6 months post-surgery, via efficacy at 2 months post-surgery. *Note*. Parameter estimates are standardized; dashed lines indicate non-significant paths. *p < 0.05, **p < 0.01.

As depicted in Figure 9, there was a negative indirect effect for optimism at 1 month post-surgery on emotion coping at 6 months post-surgery, via efficacy at 2 months post-surgery (αβ = −0.40, post. *SD* = 0.23, CI [−0.93, −0.01]) (Hypothesis 6). Similarly, there was a negative indirect effect for optimism at 1 month and 2 months post-surgery on emotion coping at 6 months post-surgery, via efficacy at 3 months post-surgery.

#### Hypothesis 7

**Figure 10:**
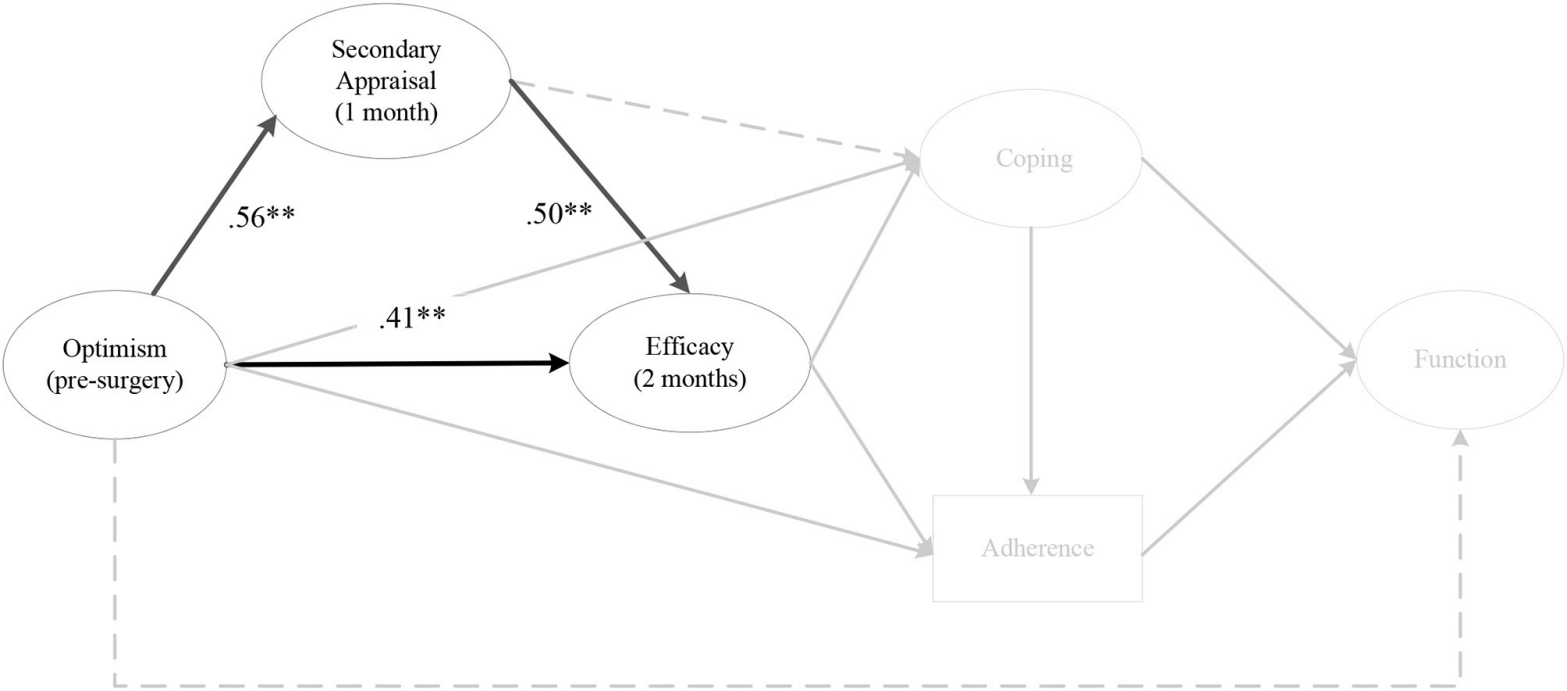
The indirect effect of pre-surgery optimism on efficacy at 2 months post-surgery, via secondary appraisal at 1 month post-surgery. *Note*. Parameter estimates are standardized; dashed lines indicate non-significant paths. **p < 0.01.

There was an indirect effect for pre-surgery optimism on efficacy at 2 months post-surgery, via secondary appraisal at 1 month post-surgery (αβ = 0.46, post. *SD* = 0.25, CI [0.12, 1.10) (Figure 10). The same effect was evident for pre-surgery optimism on efficacy at 3 months and 6 months post-surgery, via secondary appraisal at 2 months post-surgery. There was also an indirect effect for optimism at 1 month post-surgery on efficacy at 3 months and 6 months post-surgery, via secondary appraisal at 2 months post-surgery. The same relationship existed for optimism at 1 month and 2 months post-surgery on efficacy at 6 months post-surgery, via secondary appraisal at 3 months post-surgery.

#### Conceptual Model

**Figure 11:**
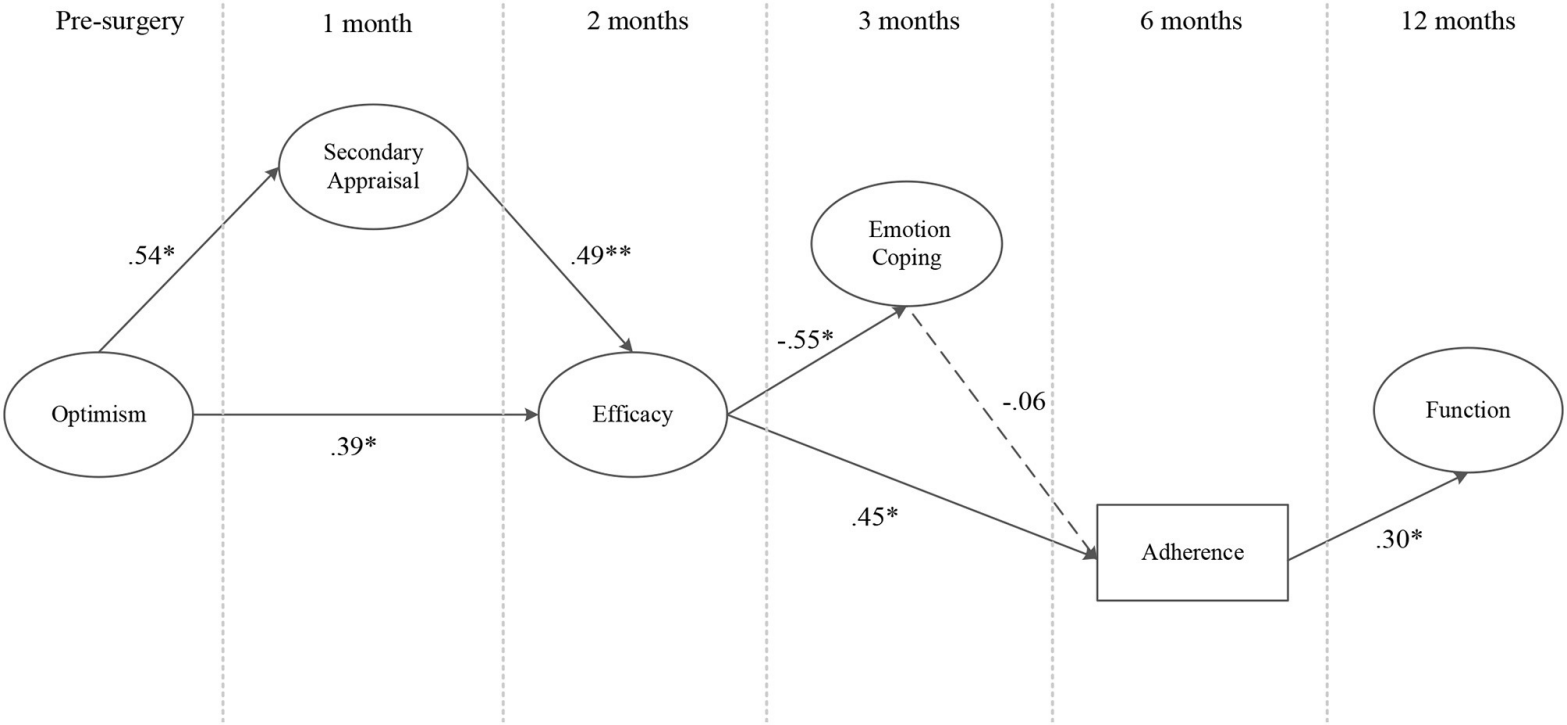
The conceptual model. *Note*. Parameter estimates are standardized and dashed lines indicate non-significant paths. *p < 0.05, **p < 0.01.

The findings from stage three of the analysis (i.e., Hypotheses 1-7) provided empirical support for the revised conceptual model in Figure 11, which was then tested in its entirety. The probability of the conceptual model, given the data, was excellent (PPP = 0.738 Δobserved and replicated χ^2^ 95% CI [−65.597, 32.991]). Two chains were estimated and within 17,000 iterations reached an appropriate convergence criteria (Muthén and Asparouhov, 2012). Visual inspection of the trace plots, and a smooth decrease in PSR development (until the last few thousand iterations were close to 1), verified support for convergence. All intended factor loadings were good (>0.42) and significant, and all cross-loadings small (< ±0.11), and non-significant.

The results of the model demonstrated pre-surgery optimism had a significant overall indirect effect on perceived knee function at 12 months post-surgery (*sum of indirect*; αβ = 0.08, post. *SD* = 0.05, CI [0.01, 0.04]), as well as a specific indirect effect through secondary appraisal at 1 month post-surgery, efficacy at 2 months post-surgery, and rehabilitation adherence at 6 months post-surgery (αβ = 0.03, post. *SD* = 0.03, CI [0.00, 0.10]). Emotion coping at 3 months post-surgery did not significantly predict rehabilitation adherence ratio at 6 months post-surgery (*p* = 0.30) as hypothesized, however, it was negatively associated with efficacy (*p* = 0.01).

## Discussion

This study extends previous research in an injury context by examining the indirect relationships between optimism, psychosocial factors and perceived knee function prior to, and throughout, the first year following ACL reconstruction; relationships that have not been previously examined despite their importance to our understanding of the complex interplay between psychosocial factors and recovery outcomes (Brewer, 2010). The findings from the third stage of the analysis (i.e., Hypotheses 1–7) provided empirical support for a number of previously untested indirect relationships, but also informed the construction of a revised and simplified conceptual model (Figure 11). Of note, secondary, as opposed to primary appraisals, were more consistently associated with dispositional optimism (the direct effects within Hypothesis 5), and consistently mediated the relationship between optimism and efficacy (Hypothesis 7). These findings support those of Chang (1998), who suggested that while optimists and pessimists might appraise the stressor (i.e., injury) similarly (primary appraisal), optimistic individuals make more positive appraisals of their coping resources (secondary appraisal). Further, these results suggest that positive secondary appraisal (i.e., inferring greater control and effectiveness over their injury) is a central mechanism by which more optimistic individuals translate their positive expectancies of a successful outcome into the conviction that they are capable of successfully executing the behavior (i.e., rehabilitation adherence) to achieve the desired outcome. With regard to coping, instrumental, palliative, and emotion-focused coping strategies demonstrated indirect effects on perceived knee function, via rehabilitation adherence (Hypothesis 3). However, emotion-focused coping was included in the revised model because of the consistent indirect effects of optimism on emotion-focused coping, via efficacy (Hypothesis 6). Put simply, optimistic individuals utilized less emotion-focused coping because they had higher levels of efficacy. These findings support those of previous studies that demonstrate negative associations between avoidance coping and optimism (e.g., Solberg Nes and Segerstrom, 2006; Carver et al., 2010; Carver and Scheier, 2014), and self-efficacy (Luszczynska et al., 2005; Schwarzer et al., 2005).

In the fourth stage of the analysis, the revised conceptual model was examined in its entirety. The findings provided empirical support for all but one of the hypothesized relationships (the path between emotion coping and rehabilitation adherence ratio failed to reach statistical significance), in addition to a multiple indirect effect. Specifically, those who were higher in dispositional optimism prior to surgery had greater perceived knee function at 12 months post-surgery via more adaptive secondary appraisals within the first month after surgery, higher levels of efficacy at 2 months after surgery, and attended more rehabilitation appointments within the first 6 months after their surgery. Similarly, those individuals with higher levels of optimism, secondary appraisals, and efficacy also utilized less emotion-focused coping strategies at 3 months post-surgery. However, in contrast to what was hypothesized, emotion-focused coping strategies at 3 months post-surgery did not demonstrate a significant negative relationship with the number of rehabilitation appointments individuals attended within the first 6 months following surgery. One possible explanation for the lack of association between coping and other indicators of recovery is that examining coping strategies within the first 3 months after ACL surgery is too early in a rehabilitation process that typically lasts between 9-12 months. Injured athletes utilize a range of coping strategies (Udry, 1997), which appear to serve different functions, at different stages within the rehabilitation process (Carson and Polman, 2010). In situations where goal-attainment is not forthcoming, such as early in ACL rehabilitation, avoidance coping strategies may have some benefit (Carson and Polman, 2010). However, a long-term reliance on these coping strategies is likely to be maladaptive to the recovery process (Carver et al., 1989; Kim and Duda, 2003). Unfortunately, our understanding of the role of psychosocial factors, such as coping, toward the end of lengthy rehabilitation periods is poorly understood, predominantly because researchers have either examined moderate and short-term injuries (e.g., Albinson and Petrie, 2003), or assessed psychosocial factors within the first 3 months following severe injuries (e.g., Chmielewski et al., 2011).

Despite the integrated model of psychological response (Wiese-Bjornstal et al., 1998), and the biopsychosocial model of injury rehabilitation (Brewer et al., 2002) hypothesizing that personality traits can influence recovery outcomes, very little research has examined this relationship (Brewer, 2010). This is surprising given that personality traits such as dispositional optimism are stable constructs (Carver et al., 2010), which, if positively associated with recovery outcomes, can supplement medical professionals existing assessment criteria in the selection of an appropriate treatment strategy (Everhart et al., 2015). While this is the first study within the psychology of sport injury literature to demonstrate the positive associations between optimism and recovery outcomes, it adds to a sizeable body of research within the health psychology literature that provides support for the effects of optimism on a range of other health outcomes (Rasmussen et al., 2009).

The second notable finding from this study was support for the mediational pathway between optimism, rehabilitation adherence, and perceived knee function. Intuitively, it is not surprising that rehabilitation adherence mediated the relationship between optimism and perceived knee function, given positive associations between optimism and rehabilitation adherence (e.g., Wadey et al., 2013), and rehabilitation adherence and recovery outcomes (e.g., Pizzari et al., 2005). However, this is one of only two studies that have examined this mediational pathway (Brewer et al., 2000), and the first to provide empirical support for it. There are two plausible explanations why this mediational pathway was supported within this study and not in (Brewer et al., 2000). Firstly, we examined the effects of a stable personality trait, optimism, as the pre-surgery predictor variable in comparison to Brewer et al. (2000) who included measures of self-motivation, social support, psychological distress, and athletic identity—all of which are more likely to fluctuate throughout the post-injury period (e.g., Tomberg et al., 2007; Brewer, 2010; Brewer et al., 2017). Secondly, in this study we included multiple assessments of perceived knee function throughout the recovery process in contrast to Brewer et al. (2000) who assessed recovery outcomes once at 6 months post-surgery; which might have been of insufficient duration for the effects of psychosocial factors and rehabilitation adherence on outcomes to emerge (Brewer et al., 2000).

The third notable finding relates to how optimistic individuals translate generalized favorable expectancies into positive health practices, including the coping strategies they adopt and the extent to which they engage with their rehabilitation. To elaborate, the findings demonstrated a multiple mediated effect between optimism, cognitive appraisal, efficacy beliefs, and coping strategies, and rehabilitation adherence. These findings have important implications for both the psychology of sport injury literature and mainstream psychology literature. Despite these constructs (optimism, cognitive appraisal, and efficacy beliefs) sharing a substantial amount of conceptual overlap (Schwarzer and Warner, 2013), they are cognitions that operate at different levels of generality (Williams, 2010). For example, while optimism is characterized by a belief that good things will happen, efficacy beliefs are competence-based, prospective, and action-related (Bandura, 1997; Schwarzer et al., 2005). Some have argued that cognitive appraisals and efficacy beliefs are the same (Lazarus and Folkman, 1984). Others, such as Bandura (1986, 1997) instead suggest that cognitive appraisals and efficacy beliefs are different cognitions that serve different functions. Our findings support Bandura's position and demonstrate that cognitive appraisal (specifically secondary appraisal) mediates the relationship between optimism and efficacy, and efficacy in turn mediates the relationship between optimism and coping strategies, and rehabilitation adherence. While it is difficult to situate these findings within the psychology of sport injury literature because of the paucity of studies which have examined cognitive appraisals, numerous studies within the health psychology literature support the mediating role of self-efficacy (Schoenthaler et al., 2009; Maeda et al., 2013; Tovar et al., 2015).

### Practical Implications

Given its relative stability and the benefits associated with an optimistic outlook (Carver et al., 2010), a pre-operative assessment of a patients optimism has the potential to help guide individualized treatment recommendations as well as identify individuals (i.e., more pessimistic people) that are at risk of compromised post-surgical recovery outcomes. These treatment recommendations should focus on how clinicians can positively influence an injured athlete's level of outcome-expectancy pre-surgery and secondary appraisals early post-surgery regarding their perceived control over, and effectiveness dealing with, their injury. Undoubtedly, this will include facilitating a patient's knowledge and understanding about their treatment, and treatment options through informational support in a bid to increase perceptions of control and autonomy (Chan et al., 2009). In a number of studies, this informational and technical support has taken the form of goal-setting (e.g., Evans et al., 2000). There is much support in the injury literature for the use of goals, specifically process and performance goals, to increase motivation and rehabilitation adherence (Gould et al., 1997; Evans and Hardy, 2002; Levy et al., 2008; e.g., Carson and Polman, 2008). While process goals can be used to focus on the execution of rehabilitation exercises in a bid to increase focus and self-confidence, performance goals, which include achieving specific standards (e.g., increased range of motion), can enhance athletes' motivation, self-confidence, and outcome-expectancy. However, a flexible approach to goal-setting should be adopted to account for the unpredictability of rehabilitation progress (e.g., Evans et al., 2000; Evans and Hardy, 2002), and goals should be regularly monitored to assess progress. That said, there may be instances where patients' post-surgical responses are considered maladaptive and are particularly debilitative. In such cases it might be necessary for athletes to seek psychological support from appropriately trained and qualified clinicians, such as sport, clinical or counseling psychologists.

### Strengths, Limitations, and Future Research

As with all forms of scientific enquiry, this study had a number of strengths and limitations. Firstly, in terms of the strengths, we adopted a number of methodological recommendations proposed by previous researchers, including the need for research to adopt (a) prospective and longitudinal research design with repeated assessments of psychosocial factors, rehabilitation adherence, and recovery outcomes (Brewer, 2010); (b) the selection of psychosocial variables guided by theory (Wadey et al., 2014); (c) an examination of factors thought to mediate the relationships between psychosocial factors and rehabilitation outcomes (Wiese-Bjornstal, 2014); (d) a homogenous sample with respect to injury type (Evans et al., 2006); and (e) the use of psychometrically sound and, where appropriate, sport injury-specific measures (Brewer and Cornelius, 2003). Indeed, the seven waves of data measured before reconstructive surgery to 1 year post-surgery, to the best of our knowledge, represents the most thorough examination in the psychology of sport injury literature to date. Furthermore, the use of BSEMs to examine the indirect relationships between optimism, psychosocial factors and recovery outcomes is another strength of this study. Bayesian estimation has only recently begun to feature in the sport psychology literature (e.g., Gucciardi and Jackson, 2015; Niven and Markland, 2016), but offers researchers the possibility of estimating more complex models that perform better with small sample sizes (Lee and Song, 2004). This is particularly important within sport injury research, which often involves a trade-off between sample size and composition (Wadey et al., 2014).

Despite these strengths, there were some limitations within the study. Firstly, the reliance on subjective assessments of recovery outcome is a limitation. Solely using self-report measures to assess the predictor (i.e., psychosocial factors) and outcome variables (i.e., perceived knee function), can increase the likelihood of shared method variance (Johnson et al., 2011). Although these concerns were reduced in the present study by temporally separating measures of the predictor and outcome variables, the potential for measurement error must be acknowledged. Another limitation relates to the measurement of rehabilitation adherence. Despite the widespread use of home-rehabilitation and adherence ratios to assess rehabilitation behaviors both have their limitations. For example, participants often over-report the extent to which they complete their home rehabilitation exercises (Brewer et al., 2004), and while rehabilitation adherence ratios provide a valuable objective indices of rehabilitation (athletes cannot adhere to a physiotherapists recommendations if they do not attend their rehabilitation sessions), they fail to account for what (e.g., how many repetitions and with what level of intensity) athletes do during the session. Instead, the inclusion of additional measures (e.g., Rehabilitation Adherence Measure for Athletic Training; Granquist et al., 2010; accelerometers; Nicolson et al., 2018), over-and-above those already employed within this study could potentially provide a more complete representation of the multidimensional nature of rehabilitation adherence. Finally, the high attrition rate and omission from the final sample of participants who did not complete all study measures beyond the initial pre-surgery ones might also be considered a limitation, despite this approach safeguarding the integrity and homogeneity of the final sample of participants and the data derived from them (i.e., minimize the error variance associated with type of injury, surgical technique, and prognosis).

Based on these findings, there are three notable areas that warrant future research attention. First, future research is needed to replicate the findings from this study and in doing so, provide additional support for the specified relationships within the conceptual model. This replication should also include the examination of the stability of this conceptual model later in the rehabilitation period, especially given the recent recommendations which suggest an athletes' return to unrestricted activity should be delayed until 2 years after ACL reconstruction to ensure baseline joint health and function (Nagelli and Hewett, 2017). Second, although this study demonstrated the positive associations between optimism and perceived knee function, future research is needed to ascertain whether these positive associations remain with more objective indices of recovery outcomes, including physical (e.g., knee strength), functional (e.g., hop tests) and biological (e.g., neurochemistry) recovery outcomes from the biopsychosocial model. This is important because the relationship between optimism and health outcomes is moderated by the nature of the outcomes assessed. That is, the effect sizes for studies using subjective measures to assess health outcomes are significantly higher than those studies using objective measures (Rasmussen et al., 2009). Therefore, a valuable contribution to the psychology of sport injury literature generally, and the propositions of the biopsychosocial model specifically, can be gleaned from future research studies that demonstrate positive associations between optimism and a range of physical and functional outcomes. Finally, the equivocal findings for coping in the present study highlight the need for more systematic coping research following sport injury. Recent research has also shown that optimistic individuals are more adept at changing their coping strategies in response to the changing nature (i.e., controllability) of the stressor (e.g., Reed, 2016). While this concept, coping flexibility, is not new, it has received increased research attention recently within the health domain (e.g., Cheng et al., 2014; Kato et al., 2019; Kroemeke, 2019), however, no studies to date have examined coping flexibility within the psychology of sport injury literature. Given that injured athletes utilize a range of coping strategies (e.g., Gould et al., 1997) that serve different functions at different stages in the rehabilitation process (e.g., Carson and Polman, 2010), this appears to be an important area of future research.

## Conclusion

A growing body of literature has highlighted the importance of psychosocial factors during the recovery process, and ultimately, on patients' recovery outcomes. Yet there has been little examination of the underlying mechanisms that influence the relationships between these constructs. The present study sought to address this limitation within the literature by adopting a research design (seven waves of data), and an emerging methodology (BSEM) in this context, to systematically examine these relationships. The findings from this study provide support for a number of previously untested hypothesized relationships within the integrated response (Wiese-Bjornstal et al., 1998) and biopsychosocial models (Brewer et al., 2002). However, they also go beyond these models by highlighting the mechanisms by which positive outcome expectancies are translated into adaptive behavioral responses such as how injured athletes cope with, and adhere to, their rehabilitation to safeguard their recovery. In doing so, we provide a conceptual model that can provide the foundations for future research moving forward.

## Data Availability Statement

Requests to access the datasets should be directed to tom.williams@southwales.ac.uk.

## Ethics Statement

The studies involving human participants were reviewed and approved by Cardiff School of Sport Research Ethics Committee at Cardiff Metropolitan University. The patients/participants provided their written informed consent to participate in this study.

## Author Contributions

TW, LE, AR, and LH were responsible for the conception and design of this longitudinal mediation study. AR, SR, and DL identified and recruited participants into the study who met the inclusion criteria. TW collected the post-surgery data, and alongside FG and LH, conducted the data analysis. TW and LE completed the final manuscript with critical revisions made initially by LH, and then AR, SR, and DL. All authors contributed to the article and approved the submitted version.

## Conflict of Interest

AR, SR, and DL were employed by the company Cardiff Sports Orthopedics LLP. The remaining authors declare that the research was conducted in the absence of any commercial or financial relationships that could be construed as a potential conflict of interest.
